# Fixing N_2_ into cyanophycin: continuous cultivation of *Nostoc* sp. PCC 7120

**DOI:** 10.1007/s00253-022-12292-4

**Published:** 2022-11-26

**Authors:** Giulia Trentin, Francesca Piazza, Marta Carletti, Boris Zorin, Inna Khozin-Goldberg, Alberto Bertucco, Eleonora Sforza

**Affiliations:** 1 Department of Industrial Engineering DII, University of Padova, Via Marzolo 9, 35131 Padua, Italy; 2 The French Associates Institute for Agriculture and Biotechnology of Drylands, Jacob Blaustein Institutes for Desert Research, Ben-Gurion University of the Negev, 8499000 Midreshet Ben-Gurion, Israel

**Keywords:** Diazotrophic, CGP, Polypeptide, Aspartic acid, Arginine

## Abstract

**Abstract:**

Two diazotrophic cyanobacteria (*Anabaena cylindrica* PCC 7122 and Nostoc sp. PCC 7120) were cultivated to produce cyanophycin, a nitrogen reserve compound, under nitrogen fixing conditions. In preliminary continuous experiments, *Nostoc* sp. was shown to be more efficient, accumulating a higher amount of cyanophycin and showing a greater capability to fix atmospheric nitrogen in the biomass (67 mg_N_ d^−1^ of fixed nitrogen per liter of culture). The operating conditions were then optimized to maximize the cyanophycin productivity: the effect of incident light intensity, residence time and nitrogen availability were investigated. Nitrogen availability and/or pH played a major role with respect to biomass production, whereas phosphorus limitation was the main variable to maximize cyanophycin accumulation. In this way, it was possible to achieve a stable and continuous production of cyanophycin (CGP) under diazotrophic conditions, obtaining a maximum cyanophycin productivity of 15 mg_CGP_ L^−1^ d^−1^.

**Key points:**

• *Diazotrophic cyanobacteria produce stable amount of cyanophycin in continuous PBR.*

• *Nostoc sp. proved to be more efficient in producing cyanophycin than Anabaena sp.*

• *P deprivation is the major variable to increase cyanophycin productivity in continuous.*

**Supplementary Information:**

The online version contains supplementary material available at 10.1007/s00253-022-12292-4.

## Introduction

Nitrogen is the second most important element for the survival of living organisms: it is a component of amino acids, enzymes, nucleic acids, peptidoglycans and is also required in photosynthetic organisms for the synthesis of chlorophyll (Stal 2015).

However, gaseous dinitrogen (N_2_) is chemically inert and therefore metabolically inaccessible (Stal 2015), thanks to the high stability of the triple bond between the two nitrogen atoms. Furthermore, to be incorporated into biological macromolecules, it must be reduced to ammonia (Berman-Frank et al. 2003). For this reason, after sulphuric acid, ammonia is the second largest synthetic inorganic chemical manufactured in the world, and is the basic building block of the world nitrogen industry (Appl 2006). It is estimated that the annual production of ammonia is 146 million tons, at an energy cost of 28 GJ per ton, exploiting approximately 1% of the global energy consumed each year (Nørskov et al. 2016). At the industrial level, ammonia is obtained by reacting N_2_ with H_2_ at high temperatures and pressures according to the Haber-Bosch process, with most plants operated at 200–400 atm and 450–600 °C (Mariani 1969).

In recent years, the interest towards bio-sustainable industrial processes able to exploit the great potential of microorganisms for obtaining food, drugs and energy has received increasing interest (Sharma et al. 2014). Bio-based processes are in fact less energy demanding and more environmentally friendly, as they operate at ambient temperature and pressure.

A few species of prokaryotic microorganisms including cyanobacteria, termed nitrogen-fixing or diazotrophic, are able to enzymatically catalyse the reaction of nitrogen gas fixation to organic molecules at ambient temperature and pressure. Therefore, they do not require the presence of a nitrogen source in the culture medium, but instead use atmospheric nitrogen to support their metabolism (Rascio and La Rocca 2013; Stal 2015). Cyanobacteria produce many commercial relevant compounds, such as phycocyanin, zeaxanthin, β-carotene, poly-hydroxy-alkanoates, proteins and PUFAs (Borowitzka 2013; Lau et al. 2015; Levasseur et al. 2020), as well as cyanophycin (CGP), a non-ribosomal synthesized polyaminoacid compound, which is interesting as source of polyaspartic acid (PASP) and arginine, replacing the standard petrochemical-based industrial products (Du et al. 2019). Usually, polyaspartic acid is synthesized by polymerization of aspartic acid or maleic anhydride. In both cases, elevated temperature (greater than 160 °C) and by-product removal are required to achieve high molecular weights and reaction yields (Adelnia et al. 2021). Amino acids as arginine, instead, are produced through protein hydrolysis, chemical synthesis, and microbiological synthesis. Specifically, most L-arginine has been produced by the direct-fermentation method from natural carbon sources (e.g. sugar, sugar syrup, glucose from tapioca or corn). Because L-arginine contains 4 atoms of nitrogen, also a source of nitrogen must be supplied, as ammonia or ammonium sulphate (Utagawa 2004).

Cyanophycin can be synthetized by both diazotrophic and non-diazotrophic filamentous and unicellular cyanobacteria, and also by some heterotrophic bacteria (Khlystov et al. 2017; Watzer and Forchhammer 2018). Cyanophycin acts as a temporary nitrogen reserve compound. In heterocystic diazotrophic cyanobacteria, the accumulation of cyanophycin is correlated to a peak of nitrogenase activity, causing the formation of dense polar nodules in the conjunction between heterocysts and adjacent vegetative cells. This position facilitates its transportation (Sherman et al. 2000). Indeed, as early as 1980, cyanophycin synthetase activity and cyanophycinase activity were measured to be 30- and 70-fold greater in heterocysts than in vegetative cells respectively, thus suggesting that cyanophycin can be rapidly polymerized and depolymerized in such cells. This means that CGP is a dynamic reservoir rather than a passive nitrogen reserve (Gupta and Carr 1981). It has been also shown that iso-aspartyl dipeptidase is preferentially expressed in vegetative cells, to allow the release of the two amino acids arginine and aspartate, once the CGP is converted into dipeptide by cyanophycinase and then transferred into the vegetative cells (Watzer and Forchhammer 2018).

The production of cyanophycin in cyanobacteria is limited by their relatively slow growth rate compared to heterotrophic bacteria and the low achievable productivities of this biopolymer (Du et al. 2019). Also transgenic plants as *Nicotiana tabacum* and *Solanum tuberosum* were used to produce cyanophycin, even if lower production yield were obtained than bacterial strains (Nausch et al. 2016). As regard the production with heterotrophic microorganisms, 970±80 mg L^-1^ and 1.5 g L^-1^ of cyanophycin were produced with *E. coli* BL21 (DE3) and *E. coli* DH1, respectively (Frey et al. 2002; Khlystov et al. 2017). Although the heterologous CGP production in bacteria could be larger than the native one (Aravind et al. 2016), the use of photoautotrophic cyanobacteria for the synthesis of biopolymers allows to develop production processes with a significantly lower impact on the environment (Watzer and Forchhammer 2018), as heterotrophic microorganisms require the presence of an organic carbon source (Ruffing and Kallas 2016) and supplementation of reduced nitrogen. In addition, photoautotrophic microorganisms accumulate cyanophycin in its native form (25–100 kDa) (Simon 1973), while heterologous systems produce smaller size cyanophycin (25–45 kDa), which can contain additional amino acid constituents as lysine (Ziegler et al. 1998; Frey et al. 2002; Steinle et al. 2008). This suggests that there are additional factors involved in the regulation of the polymer length present in native CGP accumulating microorganisms (Frommeyer et al. 2016; Watzer and Forchhammer 2018; Watzer et al. 2020).

The relevant literature about cyanophycin production by diazotrophic cyanobacteria is limited to physiological and molecular studies and cultivation in batch systems (Simon 1973; Mackerras et al. 1990a; Canizales et al. 2021). Due to the transient accumulation during the growth phases of cyanobacteria, batch systems are not suitable to assess the potential productivity of such a compound: as an example, Simon (1973) found a maximum CGP quota (7.8% DW) in the stationary phase cells. Then, when this culture was diluted, cyanophycin played its role as a transient N reserve and was completely utilized in beginning of the new growth phase. The same pattern has recently been observed by Canizales et al. (2021), who studied the accumulation of cyanophycin using urea and ammonia as nitrogen sources in *Synechocystis* sp. PCC 6803. Other studies carried out in batch systems showed that after the addition to the cultivation medium of a source of nitrogen as ammonia, it was measured a temporary increase in the CGP quota, which however then was rapidly degraded (Mackerras et al., 1990a, 1990b). So far, batch cultivation appears to be poorly efficient in boosting the CGP productivity in cyanobacteria.

Recently, Trentin et al. (2021) demonstrated that it is possible to obtain a higher and stable production of cyanophycin by continuous cultivation of the unicellular, non-diazotrophic cyanobacterium *Synechocystis* sp. PCC 6803 under balanced phosphorus limitation. Indeed, cyanobacterial growth under phosphate-limited conditions resulted in CGP accumulation (Stevens et al. 1981; Trautmann et al. 2016; Canizales et al. 2021). Stevens et al. (1981) observed by electron microscopy that as phosphate depletion proceeded, not only the number of the CGP granules per cells, but also the diameter of each granule, increased.

Concerning the possibility of continuously cultivating diazotrophic cyanobacteria, Barbera et al. (2019) obtained remarkable productivities with *Anabaena* sp. in a continuous cultivation system, showing that the growth of diazotrophic organisms can be efficient is such operating conditions.

In this work, two diazotrophic cyanobacteria were cultivated in a continuous system under N_2_ fixing conditions to assess the possible stable production of cyanophycin. In particular, it was assessed the effect of operating variables in this continuous system, such as the inlet phosphorus concentration, the incident light intensity, the residence time, and the nitrogen availability, on biomass and cyanophycin productivities. The goal is to produce biomass having specific composition and constant quality over time, obtaining high productivities and, at the same time, reducing the costs associated with the process, thus developing a system compatible with large-scale production.

## Materials and methods

### Experimental set-up

*Anabaena* sp. PCC 7122 (*Anabaena cylindrica*) and *Nostoc* sp. PCC 7120 were purchased from UTEX Culture Collection of Algae at The University of Texas at Austin (US). Cyanobacteria were maintained in diazotrophic conditions at a constant temperature of 24±1 °C in the BG11 medium (Rippka et al. 1979), modified to remove all nitrogen compounds present: the organic buffer Hepes and Ferric ammonium citrate were substituted with Sodium bicarbonate and Ferric chloride hexahydrate (FeCl_3_·6H_2_O), respectively. The final composition of the medium was reported in Table S1 in Supplementary Information. Before use, the medium was sterilized in autoclave for 20 min at 121 °C.

Experiments were carried out in a vertical flat-plate polycarbonate photobioreactor with a working volume of 150 mL (*V*_*PBR*_), an irradiated surface of 0.005 m^2^ (*A*_*PBR*_) and a thickness of 3 cm. Light (*I*_*0*_) was provided continuously by a white LED lamp. Photon Flux Density (PFD) was measured using a photoradiometer (HD 2101.1 from Delta OHM), by means of a quantum radiometric probe which quantifies the Photosynthetically Active Radiation (PAR). The mixing was ensured by both a stirring magnet placed at the bottom of the reactor and the bubbling of 1 L h^-1^ of CO_2_-air (5% v/v) mixture. The bubbling guaranteed the carbon supply as well the control of the pH within the interval 7.5–8.5, monitored daily using a Hanna portable pH-meter (code HI9124). Moreover, this configuration allows to minimize the cells adhesion to the walls so that the system can be approximated to a Continuous Stirred Tank Reactor (CSTR). In a CSTR, the specific growth rate *μ* (d^-1^) is equal to the dilution rate *D* that is the inverse of the residence time *τ* (d) (Eq. (1)). By definition, the residence time is equal to the ratio between the volume of the reactor (*V*_*PBR*_) and the volumetric flowrate (*Q*).

1 $$\mu =D=\frac{1}{\tau }=\frac{Q}{{V}_{PBR}}$$

The volume of the reactor (*V*_*PBR*_) was maintained constant thanks to an overflow pipe, which allows the output of the exhausted biomass with the same flowrate (*Q*) at which the fresh medium was pumped inside the reactor, by means of a multichannel peristaltic pump (205S/CA, Watson-Marlow Fluid Technology Group). So, by changing the residence time, i.e. changing the flowrate *Q*, it is possible to set different growth rates. With this system, after a transitory period of about three times the residence time, steady state was achieved. In this state, nutrient consumption, biomass concentration and composition remained constant until the experimental condition changed, and a new transitory period was observed. Just in case, the presence of contaminants in the reactor was checked periodically, by plating the samples in LB Petri dishes, and the culture was discarded in case of contamination. Accordingly, the productivity *P*_*i*_ (g_i_ L^-1^ d^-1^) was calculated as the ratio between the concentration of the component *i* measured at steady state (*c*_*i*_) (e.g. biomass, cyanophycin, nitrogen) and the residence time (*τ*):

2 $${P}_{i}=\frac{{c}_{i}}{\tau }$$

Steady state achievement was monitored daily through optical density measurement at 750 nm, with a UV-visible double beam spectrophotometer (UV1900, by Shimadzu, Japan). When steady state was achieved, it was kept for at least a period equal to three times the residence time, and samples of exhausted culture medium were withdrawn daily from the reactor for quantification and composition analysis. Dry cell weight (*c*_*x*_) at steady state was measured filtering under vacuum, through 0.45 μm previously dried nitrocellulose filters, which then were dried for 2 h at 105 °C in a laboratory oven. Biomass composition at steady state was characterized in terms of phosphorus, nitrogen, cyanophycin and protein internal quotas. Phosphorus and nitrogen content in the biomass were measured on centrifuged samples to remove the supernatant, at 9960 rcf (relative centrifugal force) for 10 min. The method used is an alkaline persulfate digestion (Ameel et al. 1993), followed by the quantification of released orthophosphates and nitrates. Orthophosphates are quantified following the protocols of Innamorati et al. (1990), whereas nitrates are measured with the diagnostic kit Hydrocheck Spectratest (Code 6223). Furthermore, at steady state, the PFD was measured also at the back surfaces of the PBR (*BI*) to calculate photosynthetic efficiency based on the PAR, as in Eq. (3).

3 $${\eta }_{PAR}=\frac{{c}_{x}\cdot Q\cdot LHV}{{PFD}_{abs}\cdot {E}_{P}\cdot {A}_{PBR}}$$

where *c*_*x*_ is the steady state biomass concentration, *Q* is flowrate, *PFD*_*abs*_ is the difference in the irradiance between the front (*I*_*0*_) and the back (*BI*) of the photobioreactor surface, *A*_*PBR*_ is the irradiated surface of the reactor, *E*_*P*_ is the average energy of photons (0.223 kJ mmol^-1^), and *LHV* is the Lower Heating Value of biomass (12.28 kJ g_x_^-1^), calculated with equations reported by Vardon et al. (2011) and Sung et al. (2019).

The effect of the inlet phosphorus concentration on cyanophycin productivity was investigated with both the cyanobacterial species (*Anabaena* cylindrica PCC 7122 and *Nostoc* sp. PCC 7120). The residence time (*τ*) and the incident light intensity (*I*_*0*_) were kept constant respectively at 2.3 d and 450 µmol photons m^-2^ s^-1^, according to previous literature on cyanophycin production in continuous system (Trentin et al. 2021) and on continuous cultivation of diazotrophic cyanobacteria (Barbera et al. 2019). Inlet phosphorus concentration were varied from the one commonly present in standard BG11 medium (about 5 mg_P_ L^-1^) to almost 1 mg_P_ L^-1^, modifying the concentration of potassium hydrogen phosphate (K_2_HPO_4_) in the cultivation medium. Phosphorus concentration was ascertained by measuring it both in the reactor inlet and outlet stream, with the procedure described by Innamorati et al. (1990) after biomass removal by filtration. The operating conditions are summarized in Table 1.

**Table 1:** Summary of operating condition in preliminary continuous experiments with *Anabaena cylindrica* PCC 7122 and *Nostoc* sp. PCC 7120

		Inlet phosphorus concentration (mg_P_ L^−1^)	Residence time (*τ*) (d)	Incident light intensity (*I*_*0*_) (µmol photons m^−2^ s^−1^)
Effect of the inlet phosphorus concentration	*Anabaena cylindrica* PCC 7122	5.5 ± 0.5	2.3	450
		2.8 ± 0.1		
		2.0 ± 0.1		
		1.5 ± 0.1		
		1.0 ± 0.2		
	*Nostoc* sp. PCC 7120	5.9 ± 0.1	2.3	450
		2.2 ± 0.1		
		2.0 ± 0.2		
		1.7 ± 0.1		
		1.2 ± 0.1		

A second set of experiments was carried out with *Nostoc* sp. PCC 7120 only to further test the effect of the incident light intensity, the residence time, and the nitrogen availability. The effect of the incident light intensity was evaluated using two inlet phosphorus concentrations (2.01±0.17 mg_P_ L^-1^ and 1.04±0.03 mg_P_ L^-1^) at a constant residence time of 2.3 days. When addressing the effect of the residence time, instead, reactors were illuminated continuously at 450 µmol photons m^-2^ s^-1^ and phosphorus concentration in the inlet stream was equal to 1.1±0.03 mg_P_ L^-1^. Finally, the effects of nitrogen availability and pH on the reactor productivity were addressed. To achieve a pH change, the cultivation medium was modified by removing sodium carbonate and reducing sodium bicarbonate concentration to 250 mg L^-1^. Where specified, the cyanobacteria were grown in the presence of a non-limiting source of nitrogen as NaNO_3_ (3000 mg L^-1^). In both cases, the inlet phosphorus concentration, the incident light intensity and the residence time were maintained constant respectively at 1.2±0.03 mg_P_ L^-1^, 450 µmol photons m^-2^ s^-1^ and 2.3 days. The operating conditions are summarized in Table 2.

**Table 2:** Summary of operating condition in continuous experiments with *Nostoc* sp. PCC 7120

	Inlet phosphorus concentration (mg_P_ L^−1^)	Residence time (*τ*) (d)	Incident light intensity (*I*_*0*_) (µmol photons m^−2^ s^−1^)	Other modification
Effect of incident light intensity	2.01 ± 0.17	2.3	200	-
			450	
			650	
	1.04 ± 0.03	2.3	200	-
			450	
			650	
Effect of residence time	1.1 ± 0.03	1.8	450	-
		2.3		
		3		
		4.7		
Effect of nitrogen availability and pH	1.2 ± 0.03	2.3	450	-
				low pH
				low pH + NaNO_3_

### Cyanophycin extraction and quantification

Cyanophycin extraction and quantification was done according to the methods proposed by Elbahloul et al. (2005) and Trautmann et al. (2016). The pellet of known volume of the culture was resuspended in acetone at room temperature to increase the permeability of the membranes. Then, it was washed twice with 50 mM Tris-HCl, to remove soluble proteins. To solubilize cyanophycin, 0.1M HCl was used. The solubilized cyanophycin was then precipitated using 100 mM Tris-HCl. Finally, the quantification of solubilized cyanophycin was done according to the Bradford colorimetric assay using CGP standard, isolated from *Nostoc* sp.. This extraction method ensure that the proteins were not extracted, to avoid interference in the Bradford method. The extracted cyanophycin was dried, analysed in terms of amino acid composition and used as the standard for the calibration curve.

### Analysis of aminoacid composition of cyanophycin by LC–MS/MS

The analysis of the aminoacidic composition of the extracted cyanophycin was conducted by LC-MS/MS method after acidic hydrolysis. Samples of cyanophycin were exactly weighted (5 mg) and dissolved in 10 mL of 6M HCl in sealed tubes, added of solution of gamma-aminobutyric acid (GABA) used as internal standard (IS), then nitrogen was sparged in the solution to reduce oxygen concentration and tube were hermetically closed with Teflon caps. Then material was subjected to 10 min of sonication and the mixture was heated at 100 °C for 48 h to obtain complete hydrolysis of the cyanophycin. The liquid was then subjected to vacuum evaporation for 2 h to eliminate the HCl and the volume of the liquid was then dried using gentle flow of nitrogen. Finally, the volume was adjusted to 5 mL in a volumetric flask and solution was used for the LC-MS/MS analysis.

For the analysis, a chromatographic system formed by Agilent 1260 chromatograph, with autosampler and oven column was used. As detector a Varian 500MS mass spectrometer (Ion Trap) was used operating with electrospray (ESI) operating in positive ion mode. For the detection of the target amino acids, the following transitions were selected: for aspartic acid (Asp) m/z 134 and fragment at m/z 74, for arginine (Arg) m/z 175 and fragment at 70. Calibration curves were obtained preparing different ratio of Asp, Arg and IS and correlating the ratio of amount (amount of analyte/amount of IS) and the ratio of areas (area of analyte/area of IS). Calibration curves (Y represent area ratio; x represent amount ratio) were y=3941 x +135 and y=5483 x +123. The LC separation was obtained on an Agilent Z-Hilic column (3.0 x 10 mm 2.7 µm), as mobile phases acetonitrile (A), water 0,1% formic acid (B) were used. Flow rate was 0.4 mL min^-1^. Gradient starts with 2: 98:0 % A:B isocratic for 5 min, then 10:90 % A:B at 10 minutes, 40:60% A:B at 20 min, then back to initial conditions with five minutes for equilibration. Results are reported in Fig. S1 of Supplementary Information.

### Calculation of N_2_ solubility in the culture

Aspen Plus™ process simulator (V12.1) was used to predict and to carry out sensitivity analysis on nitrogen solubility as a function of operating conditions. A flash unit operated at 24 °C and 1 atm was fed with a gaseous stream (75.2% N_2_, 20% O_2_, 4.8% CO_2_, v) and with a liquid stream with the composition of the microalgal cultivation medium. The thermodynamic model used was the Elec-NRTL, which can suitably deal with ionic species included in the cultivation medium fed to the process, and with the related chemical equilibria. It was previously validated using literature data of nitrogen solubility in water (data not shown). Table 3 reports the equilibrium and dissociation reactions considered in the simulation. The formation of solid species was neglected, as it would irrelevantly complicate the simulation. The non-condensable components (O_2_, CO_2_, and N_2_) were modelled as Henry components, i.e. their solubility was evaluated according to the Henry’s law.

**Table 3:** Equilibrium and dissociation reactions included in the global chemistry

Type	Stoichiometry
Equilibrium	${H}_{2}O+{{H}_{2}PO}_{4}^{-}\rightleftharpoons {{H}_{3}O}^{+}+{HPO}_{4}^{2-}$
	$${H}_{2}O+{HPO}_{4}^{2-}\rightleftharpoons {{H}_{3}O}^{+}+{PO}_{4}^{3-}$$
	$${H}_{3}{PO}_{4}+{H}_{2}O\rightleftharpoons {{H}_{3}O}^{+}+{{H}_{2}PO}_{4}^{-}$$
	$${H}_{2}O+{HCO}_{3}^{-}\rightleftharpoons {CO}_{3}^{2-}+{H}_{3}{O}^{+}$$
	$${2H}_{2}O+{CO}_{2}\rightleftharpoons {HCO}_{3}^{-}+{H}_{3}{O}^{+}$$
	$${2H}_{2}O\rightleftharpoons {OH}^{-}+{H}_{3}{O}^{+}$$
Dissociation	${NaHCO}_{3}\to {HCO}_{3}^{-}+{Na}^{+}$
	$${{Na}_{2}CO}_{3}\to {CO}_{3}^{2-}+2{Na}^{+}$$
	$${K}_{2}{HPO}_{4}\to {HPO}_{4}^{2-}+2{K}^{+}$$

### Statistical analysis

Statistical tests were applied to data acquired at steady state, and were conducted separately for each category of data. The existence of equal variance among data was verified with Levene’s test using a confidence level of 95%. Statistically significant differences among the data were ascertained through one-way ANOVA analysis. Grouping was done according to Tukey’s multiple comparison procedure with a 95% confidence interval. Data that do not share a letter were significantly different.

## Results

### Continuous cultivation of Anabaena cylindrica PCC 7122 and Nostoc sp. PCC 7120 to produce cyanophycin

**Fig. 1:**
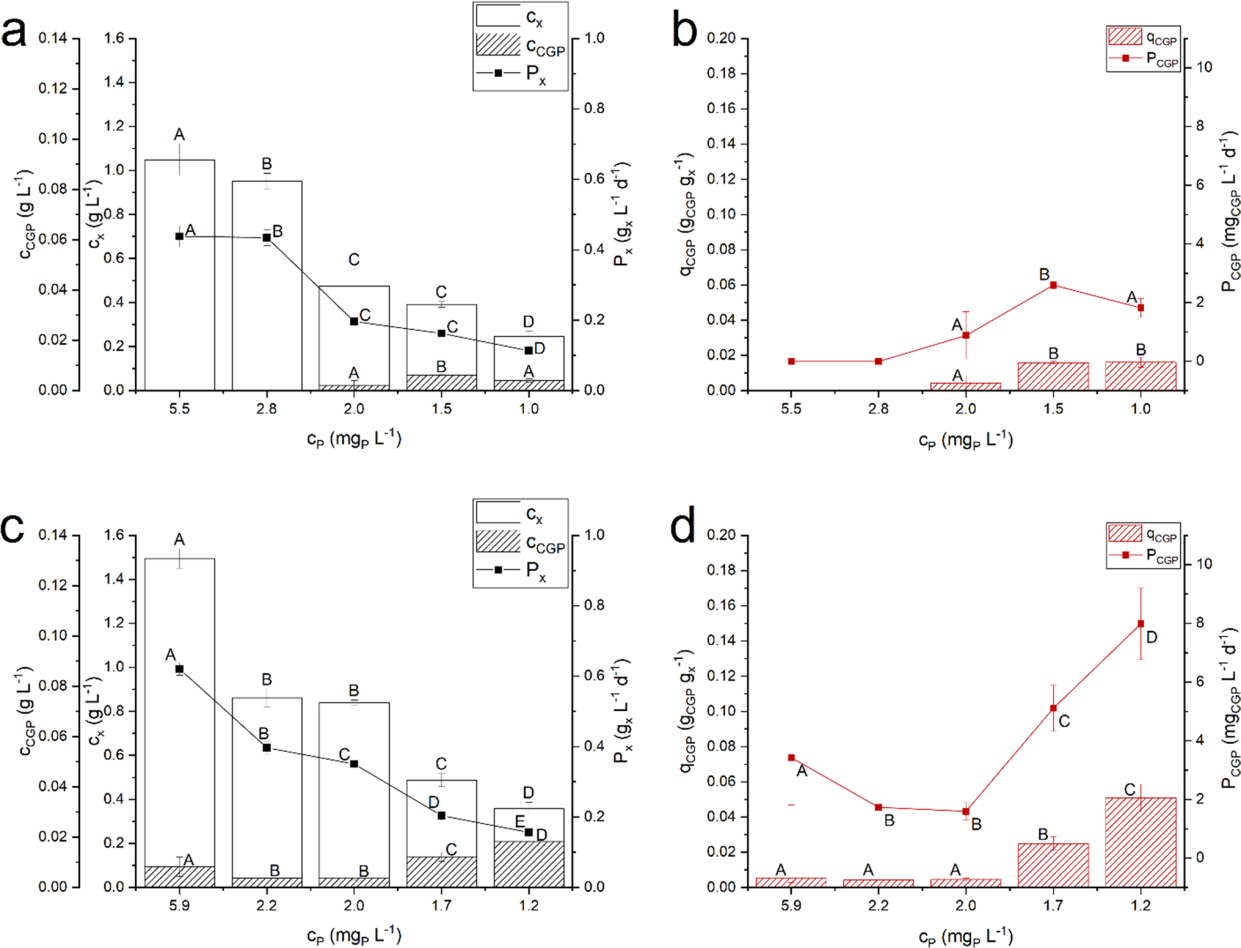
Steady state biomass concentration (*c*_*x*_), cyanophycin concentration (*c*_*CGP*_), biomass productivity (*P*_*x*_), cyanophycin quota (*q*_*CGP*_) and cyanophycin productivity (*P*_*CGP*_) as function of the inlet phosphorus concentration (*c*_*P*_) obtained with *Anabaena cylindrica* (panel *a* and *b*) and with *Nostoc* sp. PCC 7120 (panel *c* and *d*). Error bars represent the standard deviation of at least 4 samples for each steady state (*n* ≥ 4). Statistical analysis was conducted separately for each category of data. Data that do not share a letter are significantly different. Lines are just eye guides

The effect of different inlet phosphorus concentrations on the growth of two diazotrophic species was addressed, to identify which growth condition allows to obtain a higher cyanophycin productivity. The reactor was run at a residence time of 2.3 d (D=0.43 d^-1^), at a constant incident light intensity of 450 µmol photons m^-2^ s^-1^, with decreasing P concentration in the inlet, as summarized in Table 1. The results of biomass and cyanophycin concentrations and productivities were shown in Fig. 1 for both species.

For both species, the biomass concentration decreased at a decreasing inlet P concentration (Fig. 1a and 1c), as a result of nutrient limitation. However, as observed for *Synechocystis* sp. PCC 6803 and other species (Stevens et al. 1981; Trautmann et al. 2016; Trentin et al. 2021), when decreasing the inlet phosphorus concentration, the cyanophycin quota increased: the inlet concentration of phosphorus fed to the reactor had a trend that was inversely proportional to the cyanophycin quota. It should be mentioned that a commercial standard for cyanophycin quantification is not available yet. For this reason, in this work, a sample of cyanophycin from *Nostoc* sp. was produced, extracted, dried and used as a reference for the quantification of cyanophycin after the extraction. The amino acid content of the fraction extracted was analysed to verify its composition, following the protocols reported in materials and methods. It resulted that the cyanophycin produced by *Nostoc* sp. is composed by Arginine and Aspartic Acid only, as expected with the following composition: 36.2% w as Arg and 48.5% w as Asp. It should be considered, however, that the absence of a commercial compound and variation of procedures for CGP quantification is an issue when comparing our data with those from the literature, where other aminoacidic compounds are used for calibration, possibly affecting the exact quantification of the compound.

**Fig. 2:**
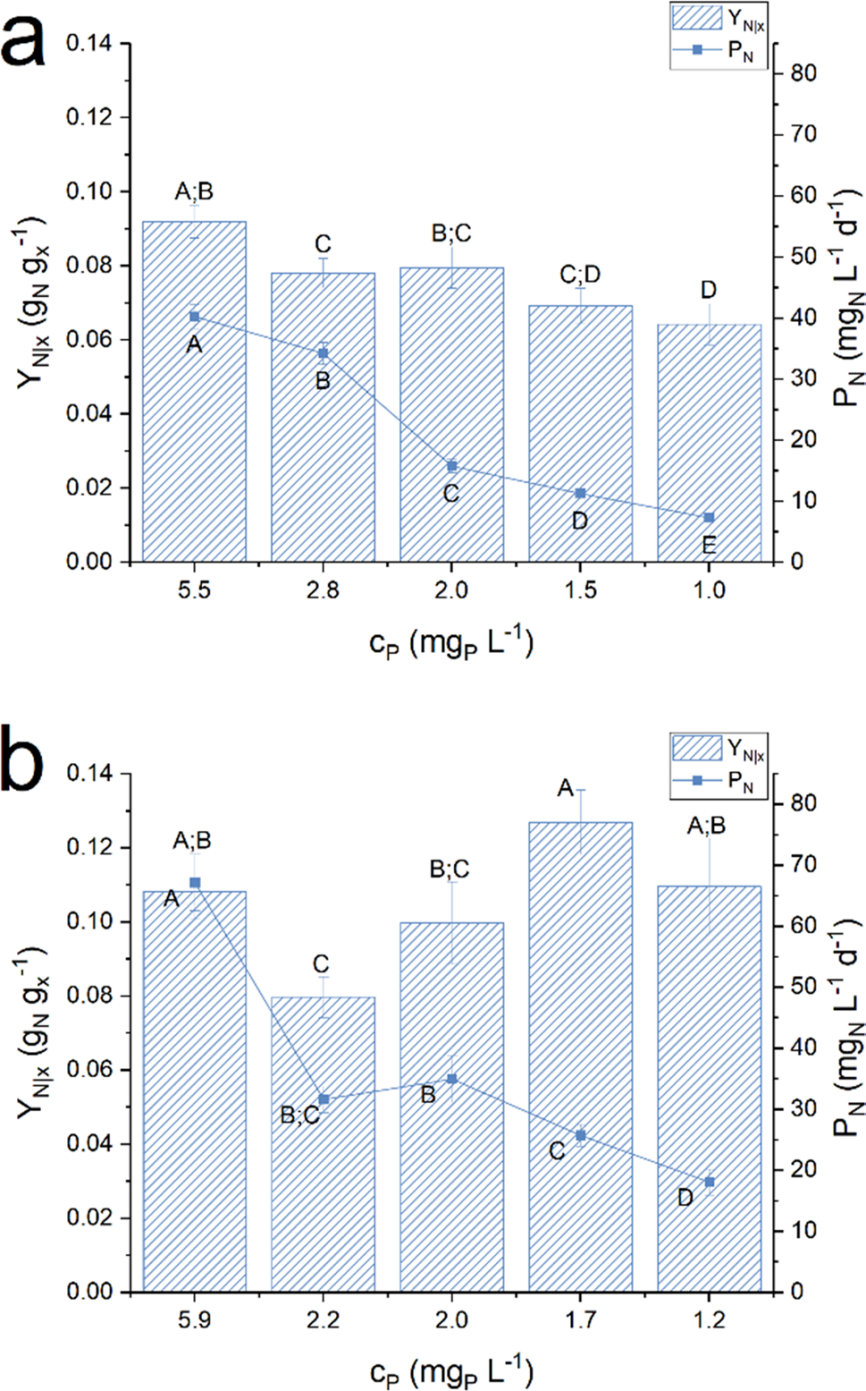
Steady-state nitrogen yield (*Y*_*N|x*_) and nitrogen fixation rate (*P*_*N*_) as function of the inlet phosphorus concentration (*c*_*P*_) obtained with *Anabaena cylindrica* (panel *a*) and with *Nostoc* sp. PCC 7120 (panel *b*). Error bars represent the standard deviation of at least 4 samples for each steady state (*n* ≥ 4). Statistical analysis was conducted separately for each category of data. Data that do not share a letter are significantly different. Lines are just eye guides

As for two tested species, *Nostoc* sp. PCC 7120 appeared the most productive, reaching a higher biomass productivity in all the conditions investigated. This could be due to a greater ability to fix atmospheric nitrogen, allowing a greater biomass productivity even in limiting conditions of phosphorus. Indeed, since nitrogen was not supplied with the culture medium, it is necessary to have a deep insight of nitrogen quota measured in the experiments. Results of nitrogen quota (Y_N|x_) and nitrogen biofixation rate (P_N_) are shown in Fig. 2.

For both species, it is clear that increasing the phosphorus in the inlet medium led to an increase in the nitrogen fixation rate. However, *Nostoc* sp. PCC 7120 achieved a greater quota of nitrogen in biomass (Fig. 2b) in each experimental condition. Thus, also the nitrogen fixation rate was greater, with a maximum value measured of 67.2 ± 4.7 mg_N_ L^−1^ d^−1^, 67% higher than that achieved with *Anabaena cylindrica* in the same experimental conditions. This value was much greater also with respect to the productivity calculated by Do Nascimento et al. (2015), that was equal to 20 mg_N_ L^−1^ d^−1^ under laboratory controlled conditions and 13 mg_N_ L^−1^ d^−1^ outdoors.

Overall, considering the preliminary results, *Nostoc* sp. PCC 7120 was more efficient both in nitrogen fixation, biomass and cyanophycin productivity. For this reason, it was selected for the subsequent studies to find the best operating conditions to maximize the cyanophycin production in continuous system.

### Effect of light intensity and residence time on cyanophycin accumulation at steady state

To find out the best operating conditions to accumulate cyanophycin in continuous cultivation of *Nostoc* sp. PCC 7120, the effect of two variables was studied: the incident light intensity and the residence time. Each one was varied, keeping the other at a constant value, as summarized in Table 2.

**Fig. 3:**
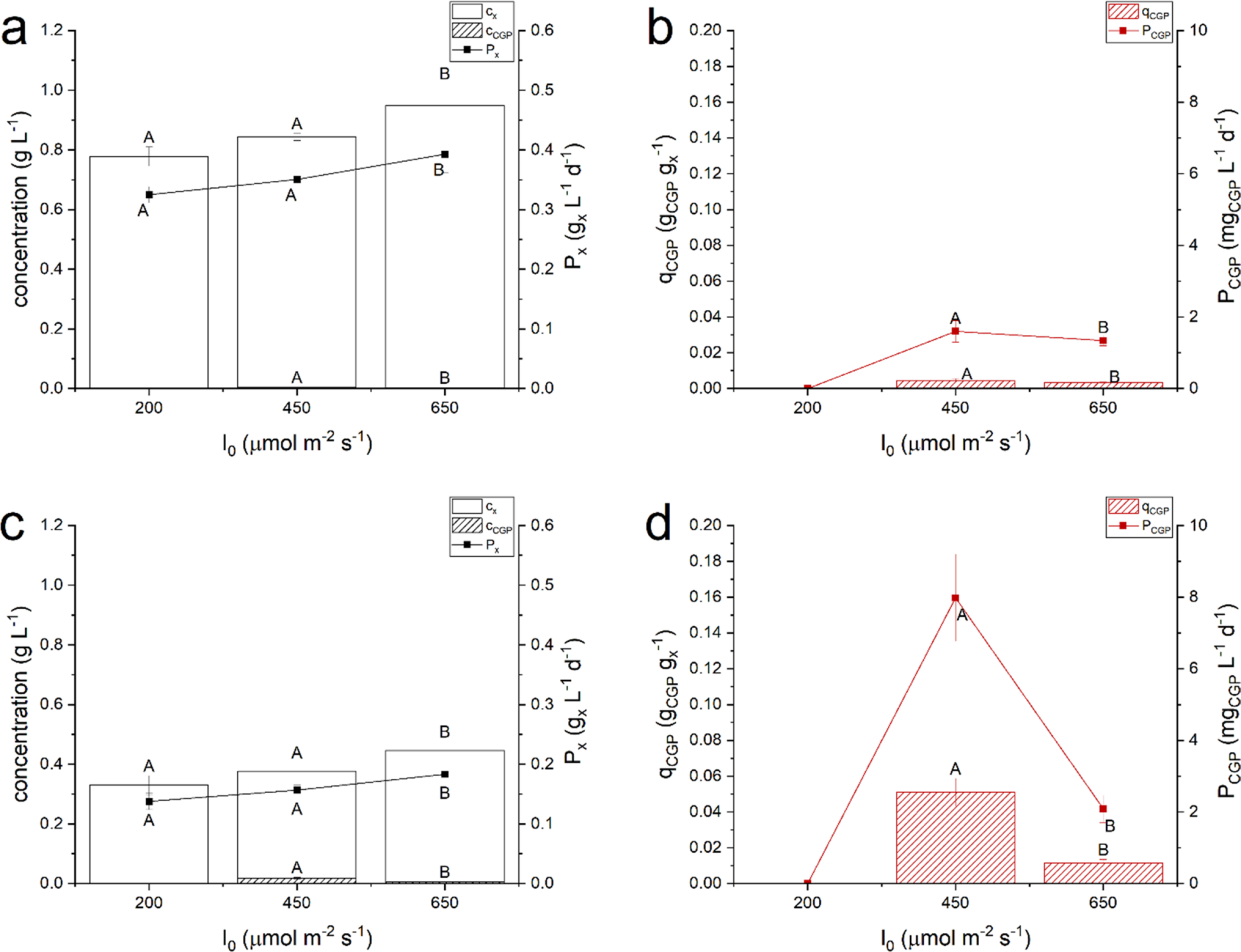
Steady state biomass concentration (*c*_*x*_), cyanophycin concentration (*c*_*CGP*_), biomass productivity (*P*_*x*_), cyanophycin quota (*q*_*CGP*_) and cyanophycin productivity (*P*_*CGP*_) as function of the incident light intensity (*I*_*0*_) with inlet P concentration equal to 2.0 ± 0.2 mg_P_ L^−1^ (panel *a* and *b*) and inlet P concentration equal to 1.0 ± 0.1 mg_P_ L^−1^ (panel *c* and *d*). Error bars represent the standard deviation of at least 4 samples for each steady state (*n* ≥ 4). Statistical analysis was conducted separately for each category of data. Data that do not share a letter are significantly different. Lines are just eye guides

Three incident light intensities were investigated: 200, 450 and 650 µmol photons m^−2^ s^−1^ using two different inlet P concentration (2.0 ± 0.2 mg_P_ L^−1^ and 1.0 ± 0.1 mg_P_ L^−1^), as at these concentrations a greater cyanophycin quota was measured in preliminary experiments. Results obtained are reported in Fig. 3.

Regardless the inlet phosphorus concentration, there was an increase in the biomass concentration as the incident light intensity increased, in line with what was reported for other microalgal species (Sforza et al. 2014). However, in this case, the biomass concentration poorly increased under higher light, possibly due to the stronger limitation caused by phosphorus depletion. Indeed, regardless the incident light intensity, also the pigment content was higher when larger amount of phosphorus was provided (2.0 ± 0.2 mg_P_ L^−1^). Regarding cyanophycin, no accumulation was observed at 200 µmol photons m^−2^ s^−1^, similarly to what was reported by Kromkamp (1987), where *Aphanocapsa* accumulated 1.5% of cyanophycin in biomass only, that was lower than that measured under high light intensities.

Based on the above results, a light intensity of 450 µmol photons m^−2^ s^−1^ and an inlet concentration of phosphorus equal to 1 mg_P_ L^−1^ were the conditions used to further study the effect of residence time (*τ*). Four were the values set (1.8, 2.3, 3.0 and 4.7 days) with results reported in Fig. S2 in Supplementary Information. Increasing the residence time, a decrease in the biomass productivity due to self-shading phenomena occurred and at *τ* = 1.8 day the highest biomass productivity was measured. However, the cyanophycin productivity had a maximum at 2.3 days, due to the higher CGP accumulation in biomass.

### Nitrogen limitation and pH affect biomass accumulation and cyanophycin production

Further experiments were carried out to ascertain the effects of nitrogen limitation and pH on cyanophycin accumulation. An incident light intensity of 450 µmol photons m^−2^ s^−1^, a residence time of 2.3 days and an inlet concentration of phosphorus equal to about 1 mg_P_ L^−1^ were the other operating conditions, as summarized in Table 2. Firstly, an experiment in the presence of sodium nitrate (3000 g L^−1^) (Table 2) was performed to assess the effect of nitrogen availability on biomass and cyanophycin productivity. The presence of nitrate ions in the medium allowed a higher biomass concentration at steady state, supporting the hypothesis that atmospheric nitrogen solubility in the medium can be limiting. The larger nitrogen availability in the culture medium caused an increase in photosynthetic yield from 0.89 ± 0.07% to 1.35 ± 0.06% (about 50%), as also observed by Fernandez Valiente and Leganes (1990) in *Nostoc* UAM 205. Interestingly, nitrate caused a decrease in the internal quota and productivity of cyanophycin, as cyanophycin seems to play a less important role in non-diazotrophic conditions (Li et al. 2001).

**Fig. 4:**
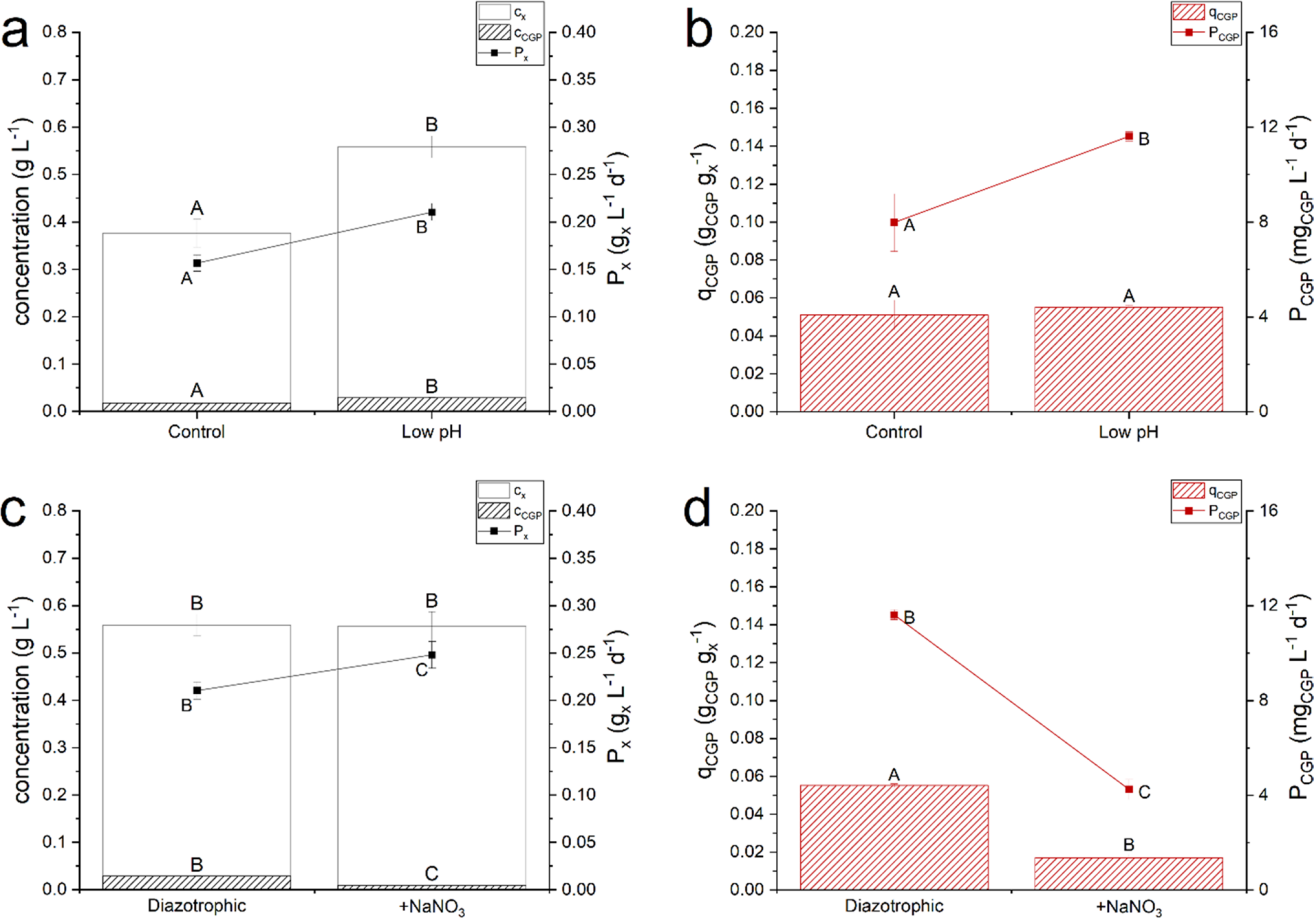
Effect of nitrogen availability and pH on *Nostoc* sp. PCC 7120. Steady state biomass concentration (*c*_*x*_), cyanophycin concentration (*c*_*CGP*_), biomass productivity (*P*_*x*_) in panel *a* and *c*; cyanophycin quota (*q*_*CGP*_) and cyanophycin productivity (*P*_*CGP*_) in panel *b* and *d*. Error bars represent the standard deviation of at least 4 samples for each steady state (*n* ≥ 4). Statistical analysis was conducted separately for each category of data. Data that do not share a letter are significantly different. Lines are just eye guides

The effect of pH was also investigated. To modify the pH, the composition of the cultivation medium was modified, removing all the sodium carbonate, and reducing up to 250 mg L^−1^ the concentration of sodium bicarbonate. Accordingly, the pH was kept at lower values, around 7–7.5, compared to the ones measured in the other experiments (at about pH = 8). Thanks to the continuous bubbling of 5% v/v of CO_2_, it is reasonable that this variation did not affect the carbon availability. However, the pH change resulted in an increased biomass production, as reported in Fig. 4. In any case, the cyanophycin internal quota was not affected by the pH-related availability of nitrogen resulting in an overall higher cyanophycin productivity. This resulted in an increased CGP productivity of 34% with respect to the control.

### Extreme phosphorus limitation to boost cyanophycin accumulation under diazotrophic conditions

**Fig. 5:**
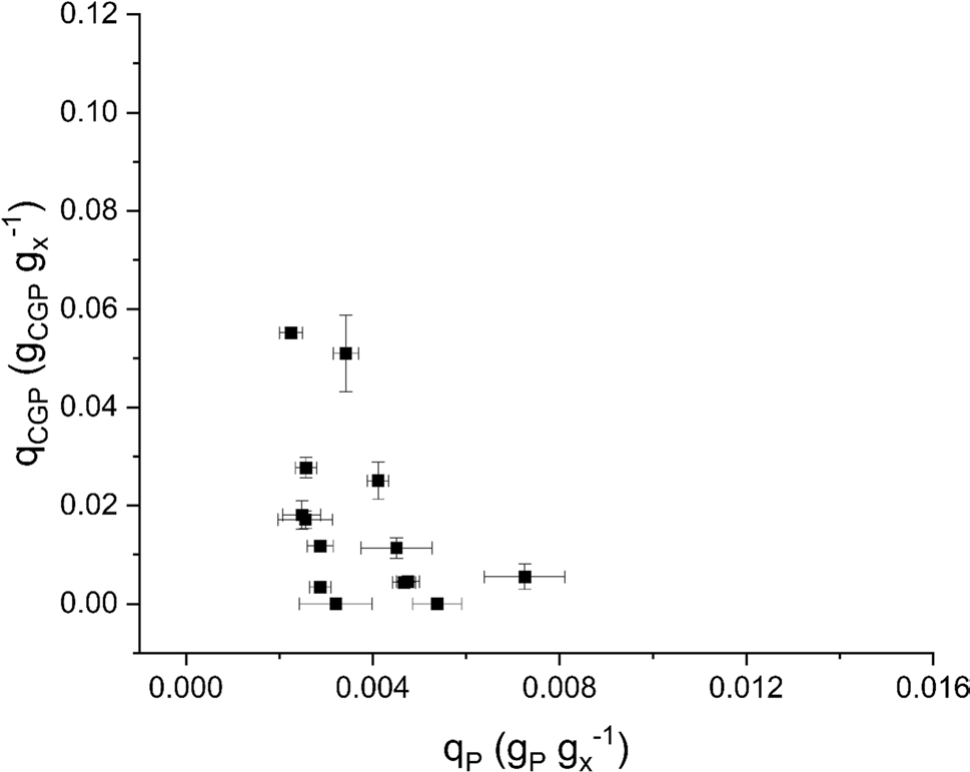
Cyanophycin quota (*q*_*CGP*_) as function of phosphorus quota (*q*_*P*_) in *Nostoc* sp. PCC 7120 cultivated in continuous systems

After evaluating the effect of light, residence time, phosphorus and nitrogen availability, a clear role of the phosphorus limitation on cyanophycin accumulation was evidenced. In fact, it was observed that the production of cyanophycin granules was strictly dependent on the concentration of phosphorus present in the culture medium. To better highlight this relation, measurement of cyanophycin quota as a function of the phosphorus quota were reported in a graph (Fig. 5), to compare the data with the observation made by Trautmann et al. (2016) and Trentin et al. (2021), both in batch and continuous system in *Synechocystis* sp.. Two variables were inversely proportional, as for *Synechocystis* sp., but with average values of internal phosphorus quota lower than those observed for such a species (0.0025 g_P_ g_x_^−1^ for *Nostoc* sp. PCC 7120, 0.004 g_P_ g_x_^−1^ for *Synechocystis* sp. PCC 6803). This suggests a possible higher specific P uptake for *Nostoc* sp. PCC 7120, with respect to other species. For this reason, a test under extreme phosphorus deprivation (0.6 mg_P_ L^−1^) was carried out.

**Fig. 6:**
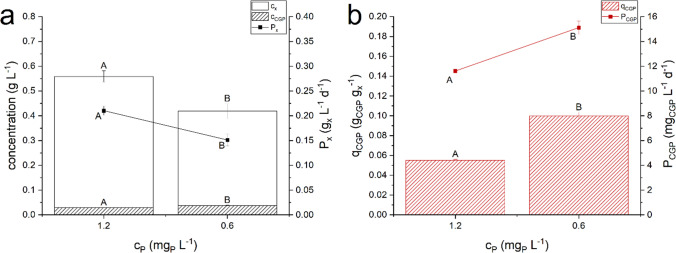
Effect of inlet phosphorus concentration on *Nostoc* sp. PCC 7120. Steady state biomass concentration (*c*_*x*_), cyanophycin concentration (*c*_*CGP*_), biomass productivity (*P*_*x*_) in panel *a*; cyanophycin quota (*q*_*CGP*_) and cyanophycin productivity (*P*_*CGP*_) in panel *b*. Error bars represent the standard deviation of at least 4 samples for each steady state (*n* ≥ 4). Statistical analysis was conducted separately for each category of data. Data that do not share a letter are significantly different. Lines are just eye guides

Results are reported in Fig. 6, showing that within further decrease of P quota, it is possible to obtain even higher amount of cyanophycin in the biomass. Indeed, a greater CGP quota was measured in the continuous system when the phosphorus quota *q*_*P*_ was lower than about 0.0025 g_P_ g_x_^−1^, and precisely equal to 0.0018 ± 0.0001 g_P_ g_x_^−1^.

As expected, decreasing the inlet phosphorus concentration, the biomass concentration decreased and *Nostoc* sp. PCC 7120 appeared strongly affected by these stressful growth conditions, leading to a more pronounced chlorosis of the biomass. On the contrary, cyanophycin quota increased, doubling its value when the phosphorus content in the biomass was reduced up to 0.18 ± 0.01% w. Accordingly, under P limitation also the cyanophycin productivity increased by the 27%.

In summary, it was possible to boost the cyanophycin productivity in a continuous system operated at steady-state, where the main variables to be managed were phosphorus supply and light.

## Discussions

The possibility of producing cyanophycin by diazotrophic cyanobacteria in continuous system was assessed by cultivating *Anabaena cylindrica* and *Nostoc* sp. PCC 7120 under progressive phosphorus limitation in the inlet. From the comparison between the two cyanobacteria, a species specificity was observed on CGP accumulation: *Anabaena cylindrica* did not produce cyanophycin under non limiting P conditions (5.5–2.8 mg_P_ L^−1^). When the inlet phosphorus concentration was reduced to 2 mg_P_ L^−1^, CGP accumulation occurred (Fig. 1b). *Nostoc* sp. PCC 7120, instead, was able to produce cyanophycin in all conditions tested, also in the cases of higher concentration of P fed (5.9–2 mg_P_ L^−1^), even though the internal quota measured was low (Fig. 1d). Moreover, when the inlet phosphorus concentration was reduced up to about 1 mg_P_ L^−1^, *Nostoc* sp. PCC 7120 was more productive reaching a two-fold cyanophycin productivity and accumulating 96% cyanophycin more than *Anabaena cylindrica*. Indeed, the contextual decrease in biomass concentration and productivity that occurred in *Nostoc* PCC 7120 under limiting conditions was not as relevant as in the case of *Anabaena cylindrica*, suggesting a higher tolerance of *Nostoc* PCC 7120 to P limitation. Accordingly, the maximum cyanophycin productivity was obtained with *Nostoc* sp. PCC 7120, also thanks to the higher biomass productivity obtained with this species and higher internal CGP quota.

Following a decrease in the inlet phosphorus concentration, the cultures changed their colour from a blue-green to a yellow-green: the synthesis of chlorophyll was reduced and the degradation of phycobiliproteins occurred, as observed macroscopically and proved by pigment content (§S1 in Supplementary Information, Table S2). As observed by Allen (1984) in batch system, this phenomenon caused the release of high quantities of nitrogen at the intracellular level, that could also explain the greater accumulation of cyanophycin measured under P limitation. Based on data of pigment content, this could also explain what was observed in our work in continuous system. All these important changes of the photosynthetic apparatus affected the light capture and, consequently, the photosynthetic yield. This was confirmed by the reduction of the photosynthetic efficiency (*η*_*PAR*_) measured in both species, and reported in Table S3 in Supplementary Information. Indeed, it was previously shown that P limitation causes a decrease in the activity of photosystem II, while there were no important differences with regard to the usual function of photosystem I (Collier et al. 1994).

Therefore, under phosphorus limitation, the cyanophycin concentration and productivity were improved and *Nostoc* sp. PCC 7120 was more effective than *Anabaena cylindrica*, in terms of both biomass and cyanophycin production. Moreover, *Nostoc* sp. PCC 7120 proved to be quite efficient as a nitrogen fixing organism, resulting in a biofixation rate equal to 67.2 mg_N_ L^−1^ d^−1^.

These preliminary results confirmed that in a continuous system the amount of phosphorus fed at the inlet was a fundamental operating variable when studying cyanophycin production, but also suggested that other ones (incident light, residence time, pH and nitrogen availability) can influence its accumulation and productivity in a continuous system. Thus, other experiments were carried out with the more promising of the two species (i.e. *Nostoc* sp. PCC 7120) to obtain a greater productivity of cyanophycin in a continuous system, exploiting atmospheric nitrogen.

The effect of other operating variables on biomass and cyanophycin accumulation was addressed, showing that lights affected the cyanophycin accumulation (Fig. 3), and specifically no cyanophycin accumulation was measured at an incident light intensity equal to 200 µmol photons m^−2^ s^−1^. Light intensity is one of the major factors in photosynthetic biomass production; however, it might impact biomass composition (Krzemińska et al. 2014), as could be seen in Table S4 in Supplementary Information, reporting the pigment content. Indeed, when cyanobacteria are exposed to high light intensities, they reduce the chlorophyll content to limit photoinhibition (Sforza et al. 2012). The level of carotenoids, on the other hand, remained almost constant: these pigments, in fact, play an important role in the photoprotection mechanism, so they are not degraded at high light intensities, as was also found by Schagerl and Müller (2006) in *Anabaena cylindrica*. Limited variation in the cellular nitrogen content was observed under different light intensities (Table S5 of Supplementary Information), suggesting a possible reallocation of intracellular nitrogen to other reserve compounds, like phycobiliproteins. In this regard, J. Wang et al. (2021) studied the variation in the content of phycobiliproteins under varying light intensity in *Dolichospermum flos-aquae*, a diazotrophic cyanobacterium, observing that pigment content was inversely related to light intensity. The photosynthetic efficiency decreased at increasing light intensity due to photosaturation and photoinhibition, which is a common trend for photosynthetic cultures (data not shown). As regard the effect of the residence time, instead, a greater accumulation of cyanophycin was found at 2.3 days.

To address the effect of nitrogen availability, *Nostoc* sp. PCC 7120 was cultivated in presence of nitrates (Fig. 4). The accumulation of cyanophycin was higher under diazotrophic condition, if compared with experiment carried out in the presence of sodium nitrate. Indeed, the addition of a source of combined nitrogen source to the culture medium tends to suppress the formation of heterocysts (Fogg 1949). As cyanophycin is mainly located at the poles of the heterocysts and in the connections between the heterocysts and the vegetative cells (Sherman et al. 2000), the reduction of its content in non-diazotrophic culture conditions may be due to the decrease in the number of heterocysts, also found by observing the filaments at the optical microscope (data not shown). Furthermore, as pointed out in the literature (Picossi et al. 2004), the expression of the genes encoding cyanophycin synthase and cyanophycinase is greater in the absence of a combined nitrogen source in the medium, both in heterocysts and in vegetative cells.

The value of pH, that may also influence nitrogen availability, was found to majorly affect biomass production, but not cyanophycin accumulation (Fig. 4). Interestingly, decreasing the pH level, the concentration and productivity of biomass were significantly increased, with comparable results between the nitrate addition and the pH control. A possible explanation could be related to the effect of pH on nitrogen solubility. A sensitivity analysis was carried out using the software AspenPlus™. Results of simulation were reported in Fig. S3 of Supplementary Information.

According to simulations, the N_2_ concentration is stable up to a pH equal to 7.75. Then, it rapidly decreases at higher pH values. Specifically, the N_2_ concentration is lowered by 8% when increasing pH value from 7 to 8.5. Thus, several factors including the effect of pH itself and the indirect effect on nitrogen solubility should be accounted for in the cultivation of cyanobacteria, where an equilibrium between CO_2_, carbonate ions and bicarbonate ions is set.

Finally, it was confirmed that P limitation is the main variable affecting the cyanophycin accumulation, and it was obtained a maximum cyanophycin productivity of 15 mg_CGP_ L^−1^ d^−1^ (Fig. 6). Thus, the internal quota of P in the biomass is the trigger for CGP accumulation. However, the relation between internal P concentration and inlet quota is species specific, as highlighted by the comparison with data for other species (Fig. 5).

## Supplementary Information

Below is the link to the electronic supplementary material.

Supplementary file1 (PDF 847 KB)

## Data Availability

The datasets generated during and/or analysed during the current study are available from the corresponding author on reasonable request.
