# Supplementary material for: Fixing N2 into cyanophycin: continuous cultivation of Nostoc sp. PCC 7120
Source: Appl Microbiol Biotechnol. 2022 Nov 26;107(1):97–110. doi: 10.1007/s00253-022-12292-4 (PMC9750909; doi:10.1007/s00253-022-12292-4)
Supplement: Supplementary file 1 — Supplementary file1 (PDF 847 KB) [file 253_2022_12292_MOESM1_ESM.pdf]

Fixing N_2 into cyanophycin: continuous cultivation of *Nostoc sp*. PCC 1720

Giulia Trentin[1], Francesca Piazza[1], Marta Carletti[2], Boris Zorin[2], Inna Khozin-Goldberg[2], Alberto Bertucco[1], Eleonora Sforza[1,*]

[1]*Department of Industrial Engineering DII, University of Padova, Via Marzolo 9, 35131 Padova, Italy*
[2]*The French Associates Institute for Agriculture and Biotechnology of Drylands, Jacob Blaustein Institutes for Desert Research, Ben-Gurion University of the Negev, Midreshet Ben-Gurion, 8499000, Israel*

Supplementary Information

Submitted to *Applied Microbiology and Biotechnology*

June 2022

[*]Corresponding author: Eleonora Sforza

e-mail: eleonora.sforza@unipd.it

Tel.: +39-0498275467; fax: +39-0498275461.

Table S1 Composition of the modified BG11 medium

**Component**	**mg L^{-1}**
Na_2Mg EDTA	2
$FeCl_3 \cdot 6H_20$	12.43
Citric acid · H_20	12
$CaCl_2 \cdot 2H_20$	72
$MgSO_4 \cdot 7H_20$	150
K_2HPO_4	30.5
H_3BO_3	5.72
$MnCl_2 \cdot 4H_20$	3.62
$ZnSO_4 \cdot 7H_20$	0.44
$CuSO_4 \cdot 5H_20$	0.16
$COCl_2 \cdot 6H_20$	0.1
$Na_2MoO_4 \cdot 2H_20$	0.78
Na_2CO_3	40
$NaHCO_3$	3000

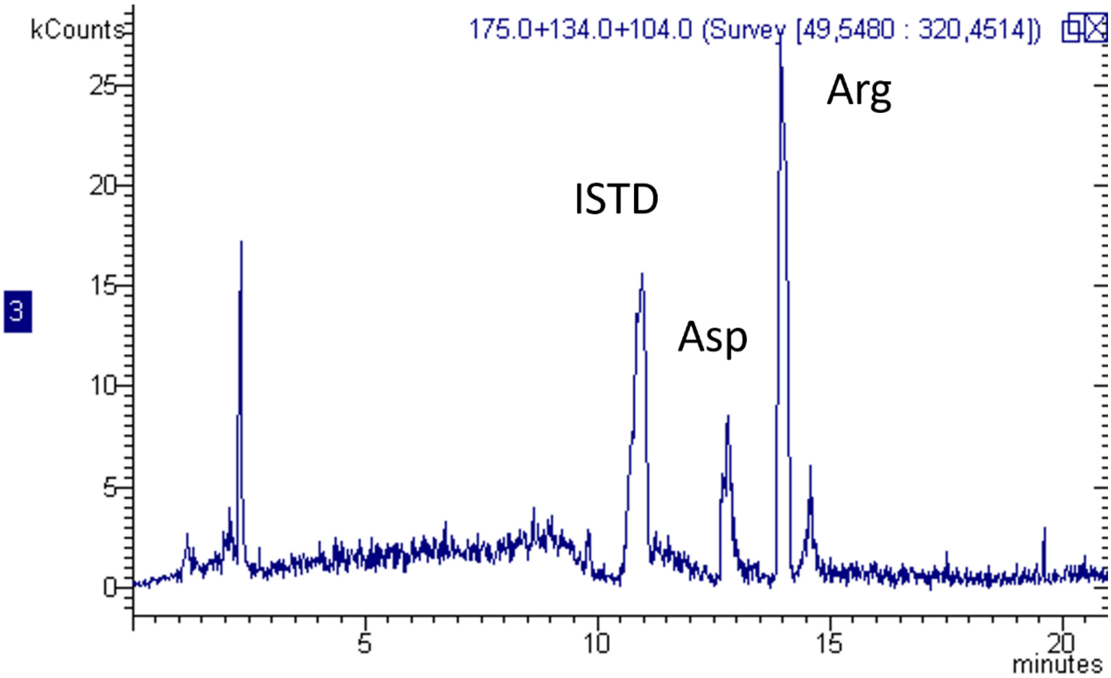

Fig. S1 Results of LC-MS/MS analysis of the extracted cyanophycin sample

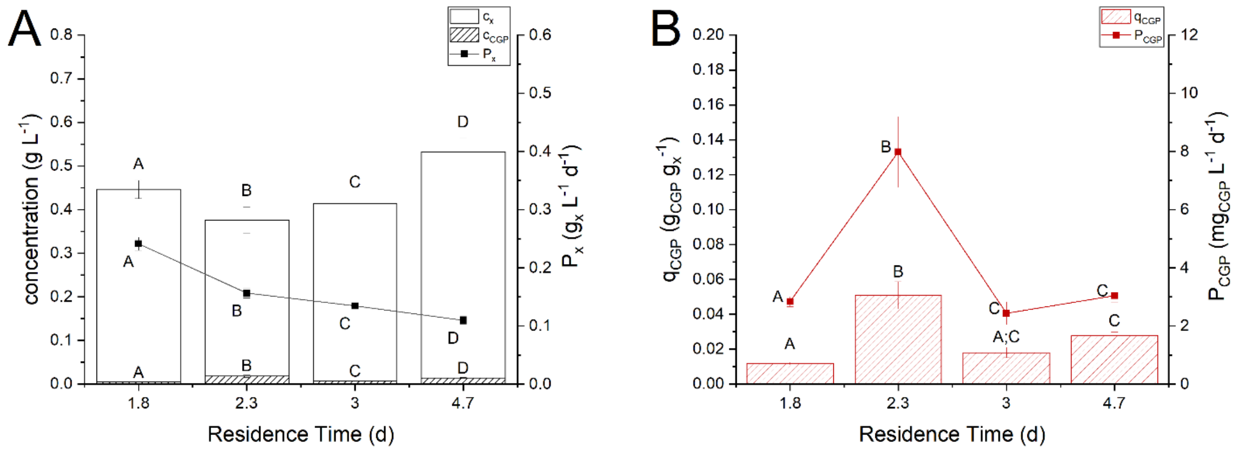

Fig. S2 Effect of residence time on *Nostoc* sp. PCC 7120. Steady state biomass concentration (c_x), cyanophycin concentration (c_{CGP}), biomass productivity (P_x) in panel A; cyanophycin quota (q_{CGP}) and cyanophycin productivity (P_{CGP}) in panel B. Error bars represent the standard deviation of at least 4 samples for each steady state (n≥4). Statistical analysis was conducted separately for each category of data. Data that do not share a letter are significantly different. Lines are just eye guides

S1. Pigment extraction and quantification

Extraction and quantification of pigment was carried out by N,N-dimethylformamide (DMF). A known volume of the culture was centrifuged at 9960 rcf for 10 min to remove the supernatant. Then, isovolume quantity of solvent was added in the dark, because once taken into solution, pigment are photosensitive. Samples were then stored in freezer for at least 48 h to ensure complete pigment extraction. The absorption spectrum on the extract was measured using DMF as reference, after a further centrifugation step. The final concentration of total chlorophyll and carotenoids was determined according to Bryant [1].

Table S2 Pigment content in biomass (mg g^{-1}) as function of the inlet phosphorus concentration obtained with *Anabaena cylindrica* PCC 7122 and with *Nostoc* sp. PCC 7120. Error represent the standard deviation of at least 4 samples for each steady state (n≥4). Data that do not share a letter are significantly different

	**Inlet phosphorus concentration (mg_P L^{-1})**	**Total chlorophyll (mg g_x^{-1})**	**Carotenoid (mg g_x^{-1})**
Anabaena cylindrica PCC 7122	5.5±0.5	9.07±0.85^{A}	1.53±0.39^{A}
	2.8±0.1	6.52±0.62^{B}	1.05±0.05$^{A;B}$
	2.0±0.1	2.60±0.27^{C}	0.73±0.01^{B}
	1.5±0.1	3.04±0.65^{C}	1.27±0.43$^{A;B}$
	1.0±0.2	2.85±0.34^{C}	0.84±0.16^{B}
Nostoc sp. PCC 7120	5.9±0.1	14.83±1.77^{A}	2.88±0.24 $^{A;B}$
	2.2±0.1	9.31±1.20^{B}	2.57±0.32 $^{A;B}$
	2.0±0.2	16.80±1.32^{A}	4.53±0.57^{C}
	1.7±0.1	9.71±1.08^{B}	3.35±0.32^{A}
	1.2±0.1	6.79±1.20^{B}	2.15±0.40^{B}

Table S3 Effect of inlet phosphorus concentration (c_P^{inlet}) on photosynthetic efficiency η_{PAR}

Anabaena cylindrica PCC 7122		*Nostoc* sp. PCC 7120	
c_P^{inlet} (mg_P L^{-1})	η_{PAR} (%)	c_P^{inlet} (mg_P L^{-1})	η_{PAR} (%)
5.5±0.5	1.89±0.13	5.9±0.1	2.65±0.08
2.8±0.1	1.93±0.07	2.2±0.1	1.77±0.08
2.0±0.1	1.17±0.19	2.0±0.2	1.65±0.02
1.5±0.1	1.04±0.04	1.7±0.1	0.97±0.06
1.0±0.2	0.82±0.08	1.2±0.1	0.89±0.07

Table S4 Pigment content in biomass (mg g^{-1}) as function of the incident light intensity (I_0) with inlet P concentration equal to 2.0±0.2 mg_P L^{-1} and inlet P concentration equal to 1.0±0.1 mg_P L^{-1}. Error represent the standard deviation of at least 4 samples for each steady state (n≥4). Data that do not share a letter are significantly different

	**Inlet phosphorus concentration (mg_P L^{-1})**	**Residence time (τ) (d)**	**Incident light intensity (I_0) (µmol photons m^{-2} s^{-1})**	**Total chlorophyll (mg g_x^{-1})**	**Carotenoid (mg g_x^{-1})**
Effect of incident light intensity	2.01±0.17	2.3	200	15.92±2.53^{A}	3.20±0.43^{A}
			450	16.80±1.32^{A}	4.53±0.57^{B}
			650	7.44±0.69^{B}	2.53±0.22^{A}
	1.04±0.03	2.3	200	9.51±0.62$^{A;B}$	2.35±0.23^{A}
			450	6.79±1.20^{A}	2.15±0.40^{A}
			650	11.39±1.48^{B}	3.44±0.37^{B}

Table S5 Effect of incident light intensity on steady state nitrogen yield $Y_{N|x}$ (g_N g_x^{-1}). Statistical analysis was conducted separately for each category of data. Error represent the standard deviation of at least 4 samples for each steady state (n≥4). Data that do not share a letter are significantly different

	**Inlet phosphorus concentration (mg_P L^{-1})**	**Residence time (τ) (d)**	**Incident light intensity (I_0) (μmol photons m^{-2} s^{-1})**	**Nitrogen yield ($Y_{N\|x}$) (g_N g_x^{-1})**
Effect of incident light intensity	2.01±0.17	2.3	200	$0.101±0.008^{A}$
			450	$0.100±0.011^{A;B}$
			650	$0.082±0.006^{B}$
	1.04±0.03	2.3	200	$0.072±0.012^{A}$
			450	$0.110±0.013^{B}$
			650	$0.107±0.005^{B}$

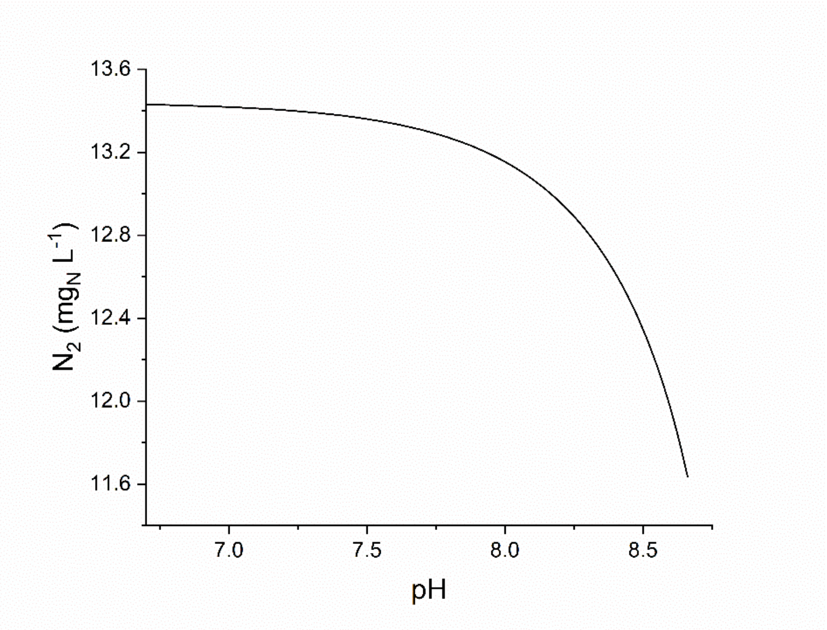

Fig. S3 Dissolved nitrogen concentration (N_2) in the culture medium as function of the pH at a constant temperature of 24°C as simulated by AspenPlus™
